# Draft genome sequences of 13 *Tenacibaculum maritimum* isolates from farmed Norwegian *Cyclopterus lumpus* (lumpfish) and *Scophthalmus maximus* (turbot)

**DOI:** 10.1128/mra.00165-24

**Published:** 2024-04-30

**Authors:** Bjørn Spilsberg, Hanne K. Nilsen, Snorre Gulla, Karin Lagesen, Duncan J. Colquhoun, Anne Berit Olsen

**Affiliations:** 1 Norwegian Veterinary Institute, Ås, Norway; 2 Norwegian Veterinary Institute Bergen, Bergen, Norway; Montana State University, Bozeman, Montana, USA

**Keywords:** tenacibaculum, mouth rot, fish farming, cleaner fish, pathology

## Abstract

Thirteen bacterial isolates of *Tenacibaculum maritimum* were sequenced and assembled. The strains were isolated from four disease outbreaks in farmed marine fish in Norway. Eight isolates were from *Cyclopterus lumpus* (lumpfish), and five were from *Scophthalmus maximus* (turbot). Overall, sequence similarity did not correlate with host species or geographic location.

## ANNOUNCEMENT

*Tenacibaculum maritimum* is associated with serious fish disease around the world and is the causative agent of tenacibaculosis (1, 2) and can be associated with massive fish mortalities and severely reduced fish welfare and health. First reported in Norway in 2015 (3), incidences of *T. maritimum* infection in this country remain rare, although this may change as *T. maritimum* appears to thrive in higher sea temperatures than those historically recorded in Norway. To illuminate the epidemiology of this potentially emerging pathogen in Norwegian aquaculture, we have sequenced the genomes of 13 *T. maritimum* isolates (Table 1).

**TABLE 1:** Assembly information for the draft genomes of 13 *T. maritimum* isolates from *Cyclopterus lumpus* and *Scophthalmus maximus*

Isolate	Disease outbreak (year of isolation)	Host	Number of predicted genes	Genome length (bp)	No. of reads	Sequencing depth (×)	GC content (%)	No. of contigs	*N*_50_ (bp)	RefSeq accession no.	SRA accession no.
NVIB-873	1 (2014)	*C. lumpus*	2,993	3,313,253	352,091	63	32.0	135	44,827	GCF_021246405.1	SRR27086638
NVIB-874	1 (2014)	*C. lumpus*	2,977	3,315,480	423,244	76	32.0	109	63,510	GCF_021246425.1	SRR27086637
NVIB-875	1 (2014)	*C. lumpus*	2,991	3,315,058	382,312	69	32.0	131	58,692	GCF_021246385.1	SRR27086633
NVIB-876	1 (2014)	*C. lumpus*	2,984	3,320,909	442,370	79	32.0	95	68,792	GCF_021246345.1	SRR27086632
NVIB-1184	2 (2015)	*C. lumpus*	2,893	3,237,057	367,396	66	31.9	91	69,629	GCF_021246325.1	SRR27086631
NVIB-1185	2 (2015)	*C. lumpus*	3,017	3,337,756	374,152	67	31.8	123	69,680	GCF_021246335.1	SRR27086630
NVIB-1498	3 (2016)	*S. maximus*	2,907	3,228,656	399,019	72	31.8	85	107,817	GCF_021246135.1	SRR27086629
NVIB-1499	3 (2016)	*S. maximus*	2,918	3,220,186	404,693	73	31.8	125	65,477	GCF_021246125.1	SRR27086628
NVIB-1500	3 (2016)	*S. maximus*	2,924	3,233,215	436,661	78	31.9	115	87,929	GCF_021246165.1	SRR27086627
NVIB-1501	3 (2016)	*S. maximus*	2,903	3,219,204	477,919	86	31.8	103	83,258	GCF_021246185.1	SRR27086626
NVIB-1502	3 (2016)	*S. maximus*	2,906	3,216,647	421,076	76	31.8	122	51,394	GCF_021246285.1	SRR27086636
NVIB-2096	4 (2017)	*C. lumpus*	3,137	3,374,869	388,654	70	31.8	208	39,310	GCF_021246085.1	SRR27086635
NVIB-2097	4 (2017)	*C. lumpus*	3,142	3,406,230	448,549	81	31.7	151	59,571	GCF_021246035.1	SRR27086634

The described study was based on material taken under routine health investigations and therefore did not require approval from the national experimental animal committee. Samples from *Cyclopterus lumpus* kidney and *Scophthalmus maximus* mouth/lip ulcerations were plated onto marine agar (Difco) under sterile conditions and incubated at 15°C for up to 7 days. Pale yellow, adherent, flat, round to ovoid colonies, consisting of filamentous, Gram-negative, non-motile rods (by phase contrast microscopy), were sub-cultured on marine agar and archived at −80°C. DNA was extracted from revived colonies on a Qiacube (Qiagen) utilizing a QIAamp DNA Mini QIAcube Kit. Sequencing libraries were generated with a Nextera XP DNA Kit (Illumina) and sequenced on a MiSeq (Illumina) with a V3 flow-cell and 300 bp paired-end chemistry. BBduk (from BBmap package v 38.18) was used to remove adapter sequences and for quality trimming, using trimq = 24 and minlen = 150. Reads were assembled with SPAdes version 3.15.3 (4) using the careful option. Error correction was performed with Pilon version 1.24 (5) with reads mapped on the assemblies with BBmap using maxindel = 80, minid = 0.95, ambiguous = toss, and killbadpairs = true. The assemblies were annotated with the NCBI prokaryotic annotation pipeline (6). Average nucleotide identity (ANI) was calculated with fastANI version 1.32 (7). The genomes (Table 1) and 24 publicly available genomes (8) were aligned with Parsnp version 1.7.4 (9) using PhiPack for recombination removal. A Maximum Likelihood phylogenetic tree was constructed with IQ-TREE version 2.2.2.7 (10) using a General time reversible (GTR) model with unequal rates and empirical base frequencies and allowing for invariant sites (11). Ultrafast bootstrapping was performed with 10,000 replicates (12). The tree was plotted with ggtree version 3.2.1 (13) and R version 4.1.2.

Thirteen *T. maritimum* isolates recovered from four disease outbreaks in different fish farming facilities on the south-western coast of Norway were sequenced (Table 1). Outbreak 1 took place in production area 1 near the city of Flekkefjord, outbreak 2 in production area 3 near Fitjar, outbreak 3 in production area 1 near Farsund, and outbreak 4 in production area 3 near the island of Stord.

**Fig 1:**
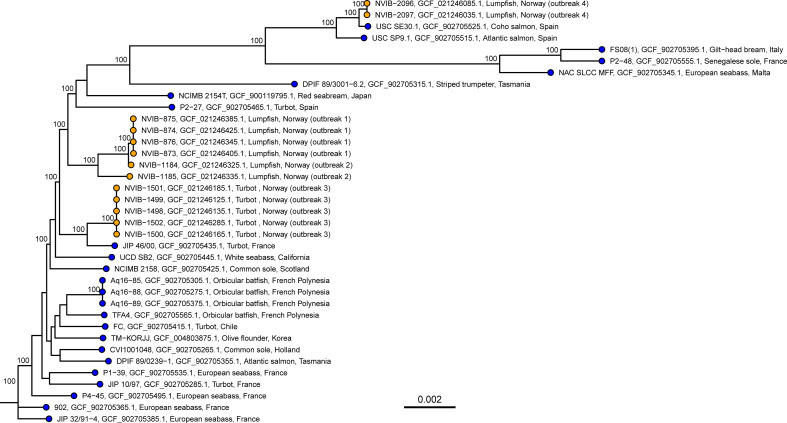
Maximum Likelihood tree of the 13 sequenced Norwegian isolates (orange tips) and 24 publicly available references (blue tips). Tip labels show isolate id, GenBank accession number, host, country of origin, and the Norwegian isolate outbreak number. The tree was constructed with IQtree using a GTR model with unequal rates and empirical base frequencies and allowing for invariant sites. The tree was based on a multiple-sequence alignment that was 2,577,608 bp long. Ultrafast bootstrap was performed with 10,000 replicates, and values ≥ 95% are shown on the nodes. The scale bar shows genetic distance (SNPs/site). The tree is rooted with JIP 32/91-4 for illustrative purposes.

The assemblies displayed ≥98.3% ANI similarity toward a *T. maritimum* reference (GCA_900119795.1) and were concluded to be *T. maritimum*. The tree (Fig. 1) demonstrates considerable diversity within *T. maritimum* as a species, and overall sequence similarity does not correlate strongly with host species or geographic location.

## Data Availability

The assemblies and raw reads from this whole-genome shotgun sequencing project have been deposited in GenBank under Bioproject PRJNA788373, with the RefSeq- and Sequence Read Archive accessions as shown in Table 1. The version described in this paper is the first version of the assemblies.
